# SDRF2GRAPH – a visualization tool of a spreadsheet-based description of experimental processes

**DOI:** 10.1186/1471-2105-10-133

**Published:** 2009-05-07

**Authors:** Hideya Kawaji, Yoshihide Hayashizaki, Carsten O Daub

**Affiliations:** 1 RIKEN Omics Science Center, RIKEN Yokohama Institute, 1-7-22 Suehiro-cho Tsurumi-ku Yokohama, Kanagawa, 230-0045 Japan

## Abstract

**Background:**

As larger datasets are produced with the development of genome-scale experimental techniques, it has become essential to explicitly describe the meta-data (information describing the data) generated by an experiment. The experimental process is a part of the meta-data required to interpret the produced data, and SDRF (Sample and Data Relationship Format) supports its description in a spreadsheet or tab-delimited file. This format was primarily developed to describe microarray studies in MAGE-tab, and it is being applied in a broader context in ISA-tab. While the format provides an explicit framework to describe experiments, increase of experimental steps makes it less obvious to understand the content of the SDRF files.

**Results:**

Here, we describe a new tool, SDRF2GRAPH, for displaying experimental steps described in an SDRF file as an investigation design graph, a directed acyclic graph representing experimental steps. A spreadsheet, in Microsoft Excel for example, which is used to edit and inspect the descriptions, can be directly input via a web-based interface without converting to tab-delimited text. This makes it much easier to organize large contents of SDRF described in multiple spreadsheets.

**Conclusion:**

SDRF2GRAPH is applicable for a wide range of SDRF files for not only microarray-based analysis but also other genome-scale technologies, such as next generation sequencers. Visualization of the Investigation Design Graph (IDG) structure leads to an easy understanding of the experimental process described in the SDRF files even if the experiment is complicated, and such visualization also encourages the creation of SDRF files by providing prompt visual feedback.

## Background

Recent technological advances have enabled a wide range of genome-scale experiments and made it easier to obtain multiple types of large-scale data focusing on a specific biological system. All of the experiments need to be combined to address specific biological questions and the series of experiments have to be designed carefully based on a particular technology's advantages and limitations so that the experiments consequently contribute to the purpose of the study. Each experimental design can be complicated, and meta-data (information about the data), as well as the actual data itself, are essential for interpreting experimental results.

In the field of microarray-based studies, MIAME (Minimum Information About a Microarray Experiment) has been widely accepted as a guideline for data submission to public repositories. MIAME requires the description of various types of information that are needed for unambiguous interpretation of the results and reproduction of the experiment [1,2]. A simple and MIAME-compliant format is MAGE-tab, which is based on a spreadsheet or a tab-delimited format [3]. This format is used for microarray and for high-throughput sequencing-based transcriptome analysis in ArrayExpress [4]. ISA-tab is a variation that extends the targeted fields by covering additional technologies [5]. One feature of these formats is a framework called SDRF (Sample and Data Relationship Format) that simply and explicitly describes the experimental process including the collection of biological materials, their preparation, and profiling protocols. This type of information is clear when a study is based on simple and typical experiments, but it can be easily missed or misunderstood when a study gets complicated or expanded to include genome-scale profiling.

A central concept underlying SDRF is the Investigation Design Graph (IDG), a directed graph that represents the experimental process [3], where each directed edge represents one step of the analysis. MAGE-tab implements the graph in a spreadsheet-based format as SDRF [3], and the implementation is used to describe 'study' and 'assay' in ISA-tab [5]. SDRF provides a practical framework for describing and exchanging information on the experimental processes, while IDG is more like a concept or idea for recognizing this information. Thus, users need to decode an SDRF file in the structure of a graph to understand the contents. The structure of the graph is obvious when the study consists of several materials and steps. However, such a structure is far from intuitive in a spreadsheet file when the study consists of many biomaterials and data objects, resulting in a single IDG with many nodes and edges. As more large-scale experiments are conducted in a study, computational support to visualize and verify SDRF files becomes essential.

Tab2mage [6] is the only tool that can handle SDRF files and it processes MAGE-tab formatted files to support microarray data submission to ArrayExpress [4]. It provides a graphical representation of a SDRF file as IDG as well as validation of the file for data submission. Here, we develop a complementary tool, SDRF2GRAPH, which focuses on the graphical representation of a wide range of SDRF files. This tool helps SDRF users, including wet scientists who may not be fully familiar with SDRF implementation, describe and exchange information about experiments. It makes the experimental process described in the SDRF files easy to understand and encourages the creation of SDRF files by providing prompt visual feedback. Our intention is not to validate a format, since specifications for MAGE-tab and ISA-tab such as acceptable column names are still being discussed [7,8], but rather to adopt less stringent rules for column names to increase its applicability to a wide range of SDRF files. To more easily facilitate the interpretation of experimental steps, we incorporate information in the graphical representation not shown previously. The enriched information in the graph clearly shows each step even if we are not familiar with the experimental design or technologies.

## Implementation

SDRF2GRAPH is written in Ruby, and the web application is implemented based on Webrick, a HTTP server bundled with Ruby. The graphical layout and image generation are realized using GraphViz [9]. The source code and its web application are available from 

## Results and discussion

### Graph structure and labels

**Figure 1:**
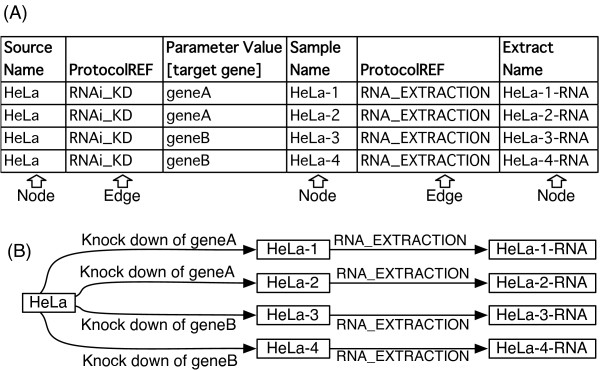
**An example of SDRF sheet and corresponding investigation design graph (IDG)**. (A) An example of SDRF spreadsheet describing knock-down experiments of two genes, gene A and B, with RNAi, where knocking-down of one gene is replicated twice (B) an example of its investigation design graph (IDG) (this graph is drawn manually.)

Firstly, we define a graphical representation to be produced by SDRF2GRAPH, since IDG was introduced to explain the concept of MAGE-tab without a formal definition [3]. Nodes, a structural component of the graph, represent biomaterials or data objects (Figure 1). All of the nodes are encoded as '~Name', '~Data', and '~File' columns (e.g. 'Source Name', 'Extract Name', and 'Image File') in SDRF, and we show objects encoded in any columns having these suffixes as nodes in the visualized graph. The only exception is the 'Array Design File' column. While this represents a data object corresponding to a microarray design, multiple microarrays with the same design are often used in a study and their graphical representation as nodes is not very informative. In fact, graphs used in the original paper on MAGE-tab [3] and its specification [7] do not show any nodes corresponding to the array design. We process this object as a parameter (see below about parameters) rather than a node. The other structural component of the graph, the direct edge, represents a protocol applied to its source node to produce its target node. It is implemented as 'Protocol REF' column in the spreadsheet (Figure 1), and we take objects encoded in this column as directed edges in the graph to be represented as described previously [3] and [7].

In addition to the structure of the graph, labels of nodes and edges show essential information of each step in the experiments. While node labels of IDG are shown in the previous works [3,7], edge labels are not incorporated. This worked fine for microarray data because there are several standard experimental designs and all of the members of this field share common knowledge about them. However, edge labels showing protocol information must be more important in a less common experimental design. Thus, we implemented an option in SDRF2GRAPH to show protocol names as edge labels. Additionally, parameters used in the protocol are required to distinguish similar but different processes as well as protocol name. When the same protocol is applied to different biomaterials with distinct parameters, the differences between them should be clear. For instance, in an RNAi perturbation study, distinct double-stranded RNAs will be transfected with the same protocol depending on the target genes. The difference between these treatments can be expressed as distinct parameter values to the same protocol (Figure 1), and parameter values are the information to distinguish these steps here. Thus, we show parameter values as well as protocol names in the edge labels. While the 'Parameter' column contains information supporting the protocol, the 'Characteristic' column contains descriptive information for the data object nodes (e.g. biomaterials, etc.). This also helps to understand what the node represents. We add this information to the node label for explicit understanding of the experimental process described in SDRF.

### User interface

**Figure 2:**
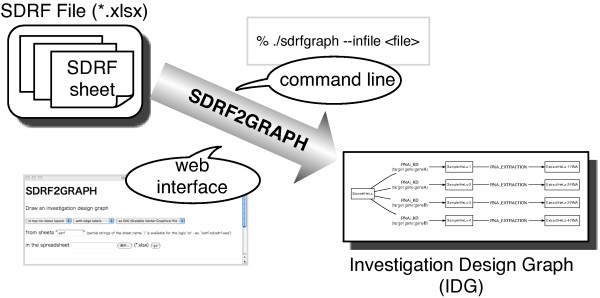
**Interface of SDRF2GRAPH**. The input of SDRF2GRAPH is one single Microsoft Excel file (Office Open XML, *.xlsx), which can contain several SDRF spreadsheets. The file is given as input via the UNIX command line or a web interface with indication of which spreadsheets are to be visualized. Subsequently, the graphical image of IDG (Investigation Design Graph) is produced as a SVG, PNG, or DOT formatted file.

To cover a wide range of potential users and usages, we designed SDRF2GRAPH to work as a stand-alone command in UNIX and as a web-based application (Figure 2). Thus, no software needs to be installed to use the latter interface. SDRF2GRAPH is designed for Microsoft Excel (Office Open XML format, *.xlsx) [10] files. These can also be used with an open-source spreadsheet software suit, OpenOffice.org, using a plug-in. This enables us to obtain immediate and frequent graphical feedback after we modify the file with spreadsheet software. Another advantage of this input over the tab-delimited flat-file is that multiple sheets can be opened in a file. A single study consisting of several steps can be divided into small pieces or layers according to arbitrary units [3], resulting in one set of SDRF spreadsheets. These spreadsheets are easily organized in one file with any spreadsheet software format. If a tool accepts only tab-delimited text files, users need to export each spreadsheet separately just to work on it. The use of spreadsheets is beneficial, particularly when a study has numerous experimental steps so that its description consists of several SDRF spreadsheets.

Many procedural steps greatly influence the size of the IDG, and a large IDG makes it difficult to follow experiments even after visualization. This can be addressed by splitting up the entire IDG into small sub-graphs corresponding to arbitrary units of experiments. Since users can define an arbitrary unit as separate spreadsheets, an option to specify the spreadsheets is provided. Visualization of the specified spreadsheets helps users when the study consists of numerous experimental steps.

### Use case (I): existing MAGE-tab and ISA-tab files

**Figure 3:**
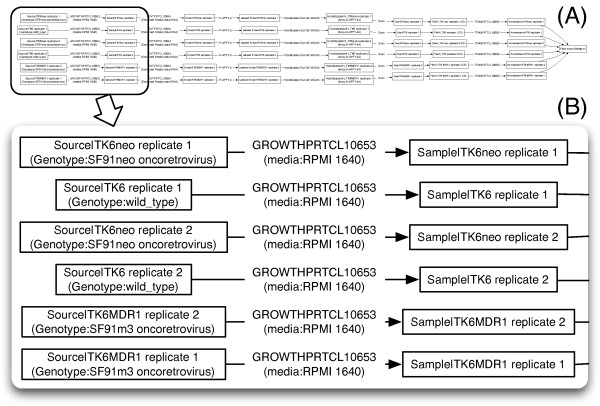
**Visualization of a MAGE-tab file**. SDRF2GRAPH visualization of a microarray study. (A) provides an overview of the graph, and (B) focuses on a part of it.

Here we show several examples of SDRF files and their graphical representations generated by SDRF2GRAPH. Firstly we show an example of a MAGE-tab file (the sample MAGE-tab file that is distributed with the specification: ArrayExpress accession: E-MEXP-428 [7,11]). This is a simple microarray study, in which three different conditions are replicated twice, and each sample is profiled with a single-color Affymetrix microarray followed by normalization. A subset of the SDRF (additional file 1; some of the columns are modified to make the graph simple, such as a node having one or less 'characteristic' and an edge having a concise 'parameter' value) is visualized by SDRF2GRAPH (Figure 3), where node characteristics ('Genotype'), edge names ('GROWTHPRTCL10653'), and their parameters ('media') are shown in the graph. The topology or overview of the graph (Figure 3(A)) shows that hybridization is performed six times, and all of the produced data are normalized and summarized in a single text file. The zoom view (Figure 3(B)) shows that the graph is self-explanatory according to the information for nodes and edges, such as genotype of devised cell line, protocol name used for cell growth, and its media.

**Figure 4:**
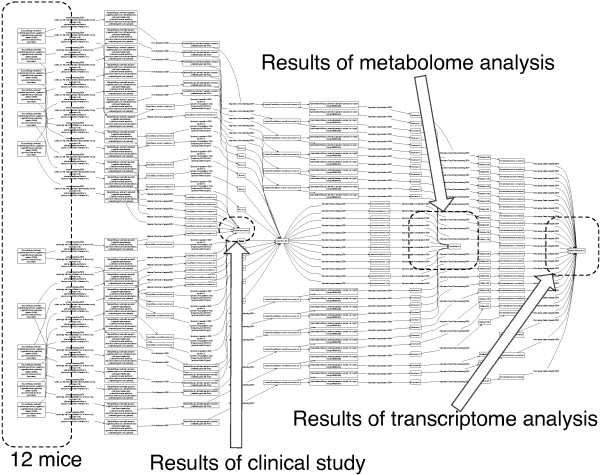
**Visualization of an ISA-tab file**. SDRF2GRAPH visualization of a study consisting of metabolome, transcriptome, and clinical chemistry analysis.

Another example is a study of the rat liver, where plasma metabonome analysis and liver transcriptome analysis are performed in combination with a clinical chemistry analysis [12]. An ISA-tab file of this study is obtained from the ISA-tab website [8] and is visualized by SDRF2GRAPH (Figure 4). This demonstrates that SDRF2GRAPH successfully visualizes the IDG structure of the study, so that we can follow the experimental process: 12 mice are fed a high-fat diet, then clinical diagnosis, metabonome and transcriptome analysis are performed. We are able to follow which mice are analyzed by which method in this visualization.

### Use case (II): FANTOM4 time course study

The examples above demonstrate the applicability of SDRF2GRAPH to existing MAGE/ISA-tab files. Here, we apply it to describe a study in our laboratory of several genome-scale experiments including novel technology to see if the tool facilitates the creation of SDRF files. DeepCAGE is a newly developed technology to quantify promoter activities by high-throughput sequencing of the mRNA 5'-end. The CAGE protocol includes a barcode-tagging process [13,14], in which a linker including a unique sequence is ligated to each RNA sample so that we can recognize the original RNA from which each 5'-end of mRNA is derived after the RNA is pooled. We had to design a unique SDRF file describing this technology.

**Figure 5:**
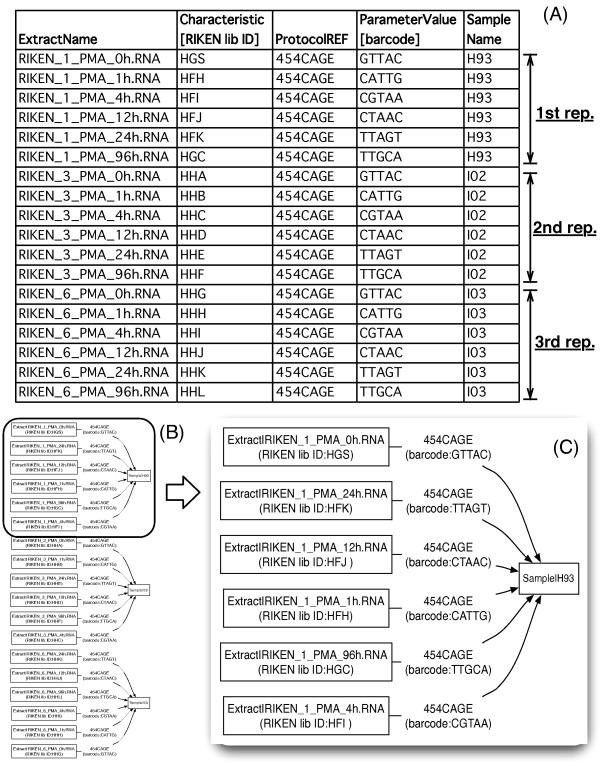
**Time series deepCAGE**. (A) A part of SDRF spreadsheet of the CAGE experiments, (B) its visualization by SDRF2GRAPH, (C) its zoom-in view of the first biological replicate.

As part of the international collaborative research activity, FANTOM4 (Functional Annotation of the Mammalian Genome 4), we applied the deepCAGE technology to a human macrophage cell line over a time course to elucidate the transcription regulatory network behind human macrophage differentiation [15]. Six time points were profiled with three biological replicates, and each time point was tagged with a different barcode and pooled per replicate. An SDRF spreadsheet corresponding to a part of these CAGE experiments, Figure 5(a), is visualized by SDRF2GRAPH (Figures 5(b) and 5(c)). The graph topology (Figure 5(b)) clearly shows three replicates, where the samples derived from different time points were pooled per replicates. A closer look at the first replicate (Figure 5(c)) shows that distinct barcodes were used for each time point.

The same samples were profiled with a conventional microarray, and the same time points (but different samples) were subjected to ChIP/chip analysis. The entire experiment consisted of several steps, and its corresponding SDRF file became quite large (additional file 2). SDRF2GRAPH visualization (additional file 2), helped our description and we received rapid feedback on the experimental design. We were able to look at connectivity and examine the replicates. (i) Connectivity: inconsistencies of node names were introduced several times in the editing step, resulting in a disconnected graph. For example, we started from a spreadsheet to describe a small piece of experiments, then expanded the SDRF by adding spreadsheets. During the expansion and repetitive revision of each sheet, we needed to go back and forth between the distinct sheets, resulting in inconsistent node names between the spreadsheets. (ii) Replicates: we used multiple types of technologies to characterize one model system with biological and technical replicates, and the wrong number of replicates was introduced several times. This was caused by incorrectly copying and pasting of rows to create rows similar to existing ones. Prompt visual feedback of the edited SDRF file made it easy to examine the graph topology and we could identify such mistakes with less effort.

### SDRF2GRAPH's advantages and limitations

A consistent description of complex experimental situations is important especially in the light of recently emerging technologies and ideas that enable us to simultaneously characterize various experimental aspects of biological material in a genome-wide and innovative way. SDRF provides a practical framework to represent such complicated experimental setups and steps, and Tab2mage [6] was the first and is the only available tool to support this framework. One of the bundled scripts, expt_check.pl, provides a functionality to visualize SDRF files, and its visualization based on GraphViz [9] helps to understand the descriptions. However, the software has two limitations: (1) it requires local installation, and (2) only a text file can be input. The former restricts the utility of SDRF files, especially for non-experts in data annotation in this field, although one of its elegant features is a simple spreadsheet-based framework. The latter limitation does not facilitate the use of multiple sheets to represent a single experiment, which is an indispensable feature of SDRF to describe large and complex experiments. SDRF2GRAPH addresses these two points by providing a representation consistent with Tab2mage [6], which is widely accepted. In contrast, SDRF2GRAPH does not offer any other functionality as implemented in Tab2mage [6], such as validation of data files, conversion to MAGE-ML [16], and other support for data submission. For the submission of microarray data to ArrayExpress for example [4], Tab2mage [6] is more suitable than SDRF2GRAPH.

The release of Isacreator [17] has been announced recently. Though the software must be installed, it does support the creation of SDRF files and their visualization with its own graphical interface. This approach will make it easier to generate complete files with rigid structures and ontologies; this is particularly beneficial for data submission to public repositories after data assembly and analysis. In contrast, SDRF2GRAPH focuses on visualization and the added benefit that no installation is required and users can create data files by themselves using their favorite software (e.g. Microsoft Excel or Openoffice.org calc).

Although SDRF provides a practical open framework, Tab2mage [6] has so far been the only available implementation supporting the format. SDRF2GRAPH promotes the applicability of the SDRF format by complementing the functionality of existing tools for the scientific community.

## Conclusion

We developed a new tool, SDRF2GRAPH, to visualize an SDRF file describing experimental steps (additional file 4). We demonstrated that it is applicable to a wide range of SDRF files, from MAGE-tab files describing transcriptome analysis to ISA-tab files describing a study consisting of multiple omics-scale technologies. It facilitates the description of experiments using various genome-scale technologies. Furthermore, it aids in the interpretation of existing SDRF files and can be used to create files for which templates do not exist. As the tool makes it easy to quickly create SDRF files describing a study, it will facilitate internal communication within large complex studies as well as formal submission of data to public repositories.

## Availability

- Project name: sdrf2graph

- Project homepage: 

- Operating system: UNIX or GNU/Linux

- Programming language: Ruby

- Other requirements: GraphViz , ruby-zip 

- License: Ruby's license

## Abbreviations

SDRF: Sample and Data Relationship Format; IDG: Investigation Design Graph; MIAME: Minimum Information About a Microarray Experiment; FANTOM: Functional Annotation of the Mammalian Genome.

## Authors' contributions

HK wrote the code of the software and the manuscript. CD and YH were involved in supervision.

## Supplementary Material

Additional file 1 **An example of SDRF file**. A part of MAGE-tab file, E-MEXP-428, which is used in Figure 3. Click here for file

Additional file 2 **An SDRF file for FANTOM4 data**. an SDRF file for the FANTOM4 data. Click here for file

Additional file 3 **SDRF2GRAPH representation for the FANTOM4 data**. SDRF2GRAPH output of the additional file 2. Click here for file

Additional file 4 **SDRF2GRAPH source code**. The source code of SDRF2GRAPH. Click here for file
