# Supplementary material for: SDRF2GRAPH – a visualization tool of a spreadsheet-based description of experimental processes
Source: BMC Bioinformatics. 2009 May 7;10:133. doi: 10.1186/1471-2105-10-133 (PMC2689195; doi:10.1186/1471-2105-10-133)

KD series 1

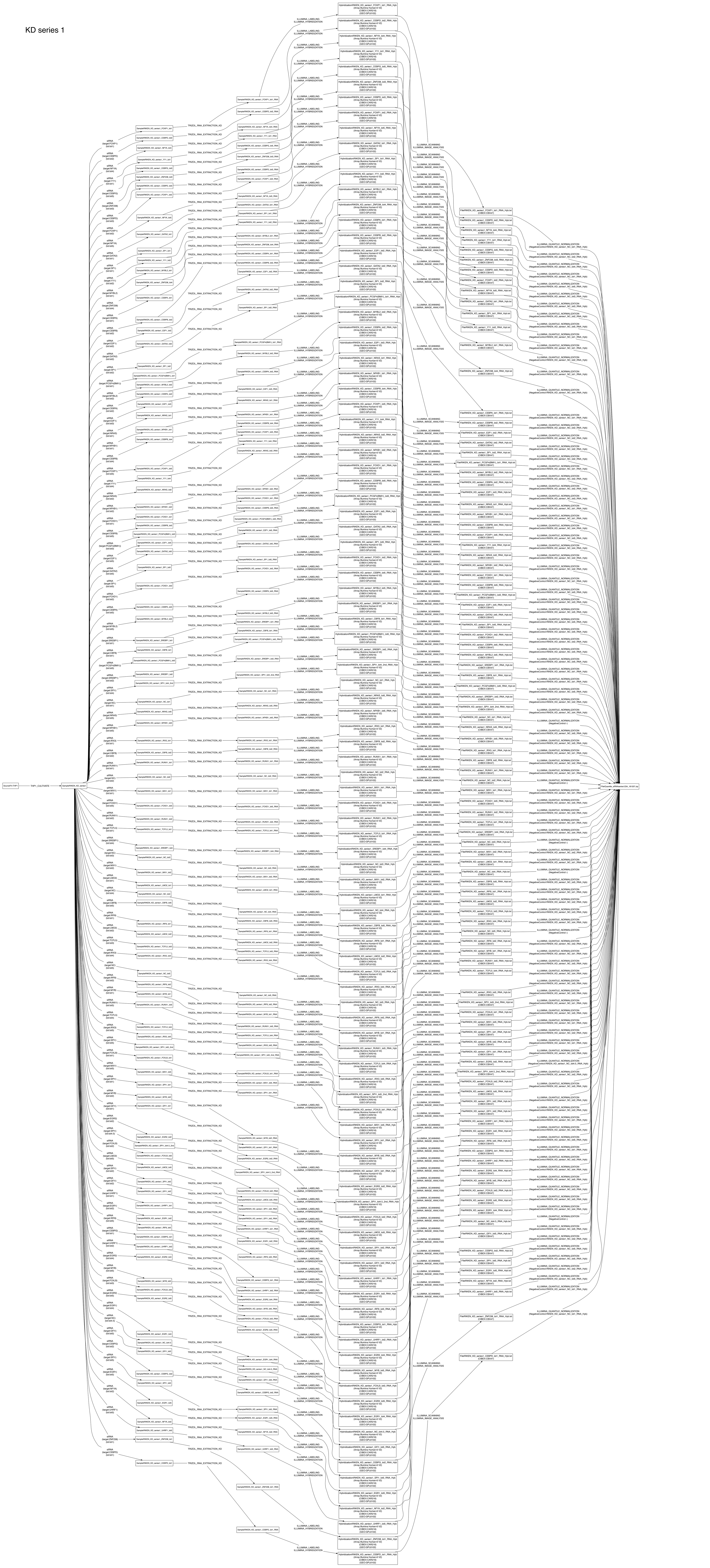

KD series 2

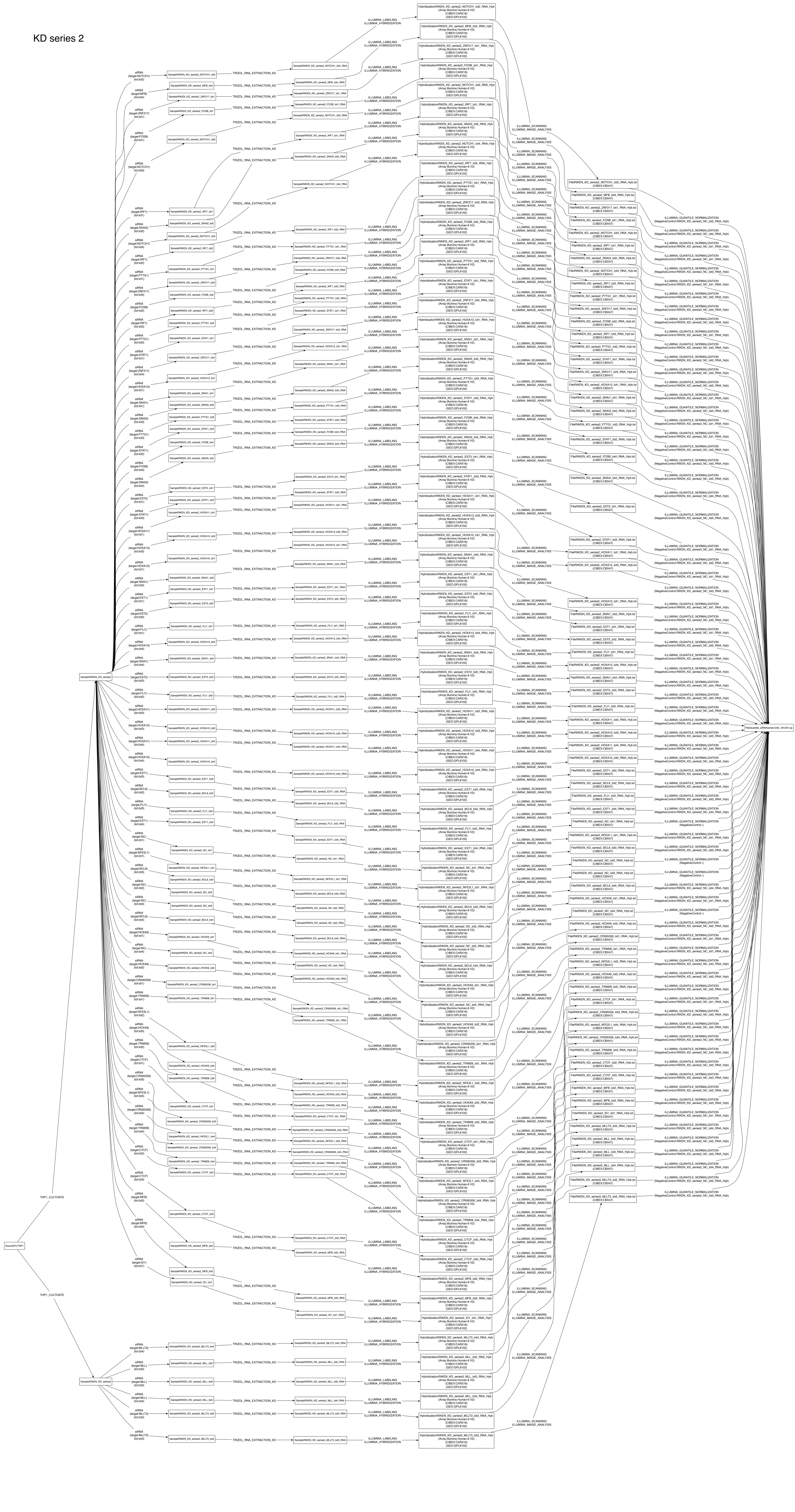

THP1_CULTIVATE
TRIZOL_RNA_EXTRACTION_KD
ILLUMINA_LABELING
ILLUMINA_HYBRIDIZATION
ILLUMINA_SCANNING
ILLUMINA_IMAGE_ANALYSIS
ILLUMINA_QUANTILE_NORMALIZATION

KD series 3

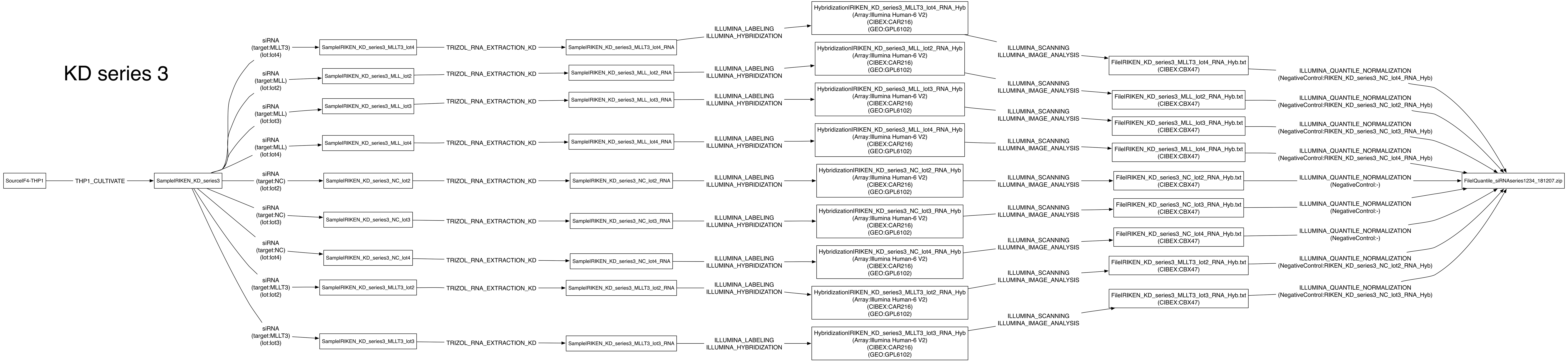
SourceIF4-THP1
THP1_CULTIVATE
SampleIRIKEN_KD_series3
siRNA (target:MLLT3) (lot:lot4)
siRNA (target:MLL) (lot:lot2)
siRNA (target:MLL) (lot:lot3)
siRNA (target:MLL) (lot:lot4)
siRNA (target:NC) (lot:lot2)
siRNA (target:NC) (lot:lot3)
siRNA (target:NC) (lot:lot4)
siRNA (target:MLLT3) (lot:lot2)
siRNA (target:MLLT3) (lot:lot3)
SampleIRIKEN_KD_series3_MLLT3_lot4
SampleIRIKEN_KD_series3_MLL_lot2
SampleIRIKEN_KD_series3_MLL_lot3
SampleIRIKEN_KD_series3_MLL_lot4
SampleIRIKEN_KD_series3_NC_lot2
SampleIRIKEN_KD_series3_NC_lot3
SampleIRIKEN_KD_series3_NC_lot4
SampleIRIKEN_KD_series3_MLLT3_lot2
SampleIRIKEN_KD_series3_MLLT3_lot3
TRIZOL_RNA_EXTRACTION_KD
SampleIRIKEN_KD_series3_MLLT3_lot4_RNA
SampleIRIKEN_KD_series3_MLL_lot2_RNA
SampleIRIKEN_KD_series3_MLL_lot3_RNA
SampleIRIKEN_KD_series3_MLL_lot4_RNA
SampleIRIKEN_KD_series3_NC_lot2_RNA
SampleIRIKEN_KD_series3_NC_lot3_RNA
SampleIRIKEN_KD_series3_NC_lot4_RNA
SampleIRIKEN_KD_series3_MLLT3_lot2_RNA
SampleIRIKEN_KD_series3_MLLT3_lot3_RNA
ILLUMINA_LABELING ILLUMINA_HYBRIDIZATION
HybridizationIRIKEN_KD_series3_MLLT3_lot4_RNA_Hyb (Array:Illumina Human-6 V2) (CIBEX:CAR216) (GEO:GPL6102)
HybridizationIRIKEN_KD_series3_MLL_lot2_RNA_Hyb (Array:Illumina Human-6 V2) (CIBEX:CAR216) (GEO:GPL6102)
HybridizationIRIKEN_KD_series3_MLL_lot3_RNA_Hyb (Array:Illumina Human-6 V2) (CIBEX:CAR216) (GEO:GPL6102)
HybridizationIRIKEN_KD_series3_MLL_lot4_RNA_Hyb (Array:Illumina Human-6 V2) (CIBEX:CAR216) (GEO:GPL6102)
HybridizationIRIKEN_KD_series3_NC_lot2_RNA_Hyb (Array:Illumina Human-6 V2) (CIBEX:CAR216) (GEO:GPL6102)
HybridizationIRIKEN_KD_series3_NC_lot3_RNA_Hyb (Array:Illumina Human-6 V2) (CIBEX:CAR216) (GEO:GPL6102)
HybridizationIRIKEN_KD_series3_NC_lot4_RNA_Hyb (Array:Illumina Human-6 V2) (CIBEX:CAR216) (GEO:GPL6102)
HybridizationIRIKEN_KD_series3_MLLT3_lot2_RNA_Hyb (Array:Illumina Human-6 V2) (CIBEX:CAR216) (GEO:GPL6102)
HybridizationIRIKEN_KD_series3_MLLT3_lot3_RNA_Hyb (Array:Illumina Human-6 V2) (CIBEX:CAR216) (GEO:GPL6102)
ILLUMINA_SCANNING ILLUMINA_IMAGE_ANALYSIS
FileIRIKEN_KD_series3_MLLT3_lot4_RNA_Hyb.txt (CIBEX:CBX47)
FileIRIKEN_KD_series3_MLL_lot2_RNA_Hyb.txt (CIBEX:CBX47)
FileIRIKEN_KD_series3_MLL_lot3_RNA_Hyb.txt (CIBEX:CBX47)
FileIRIKEN_KD_series3_MLL_lot4_RNA_Hyb.txt (CIBEX:CBX47)
FileIRIKEN_KD_series3_NC_lot2_RNA_Hyb.txt (CIBEX:CBX47)
FileIRIKEN_KD_series3_NC_lot3_RNA_Hyb.txt (CIBEX:CBX47)
FileIRIKEN_KD_series3_NC_lot4_RNA_Hyb.txt (CIBEX:CBX47)
FileIRIKEN_KD_series3_MLLT3_lot2_RNA_Hyb.txt (CIBEX:CBX47)
FileIRIKEN_KD_series3_MLLT3_lot3_RNA_Hyb.txt (CIBEX:CBX47)
ILLUMINA_QUANTILE_NORMALIZATION (NegativeControl:RIKEN_KD_series3_NC_lot4_RNA_Hyb)
ILLUMINA_QUANTILE_NORMALIZATION (NegativeControl:RIKEN_KD_series3_NC_lot2_RNA_Hyb)
ILLUMINA_QUANTILE_NORMALIZATION (NegativeControl:RIKEN_KD_series3_NC_lot3_RNA_Hyb)
ILLUMINA_QUANTILE_NORMALIZATION (NegativeControl:RIKEN_KD_series3_NC_lot4_RNA_Hyb)
ILLUMINA_QUANTILE_NORMALIZATION (NegativeControl:-)
ILLUMINA_QUANTILE_NORMALIZATION (NegativeControl:-)
ILLUMINA_QUANTILE_NORMALIZATION (NegativeControl:-)
ILLUMINA_QUANTILE_NORMALIZATION (NegativeControl:RIKEN_KD_series3_NC_lot2_RNA_Hyb)
ILLUMINA_QUANTILE_NORMALIZATION (NegativeControl:RIKEN_KD_series3_NC_lot3_RNA_Hyb)
FileIQuantile_siRNAseries1234_181207.zip

KD series 4

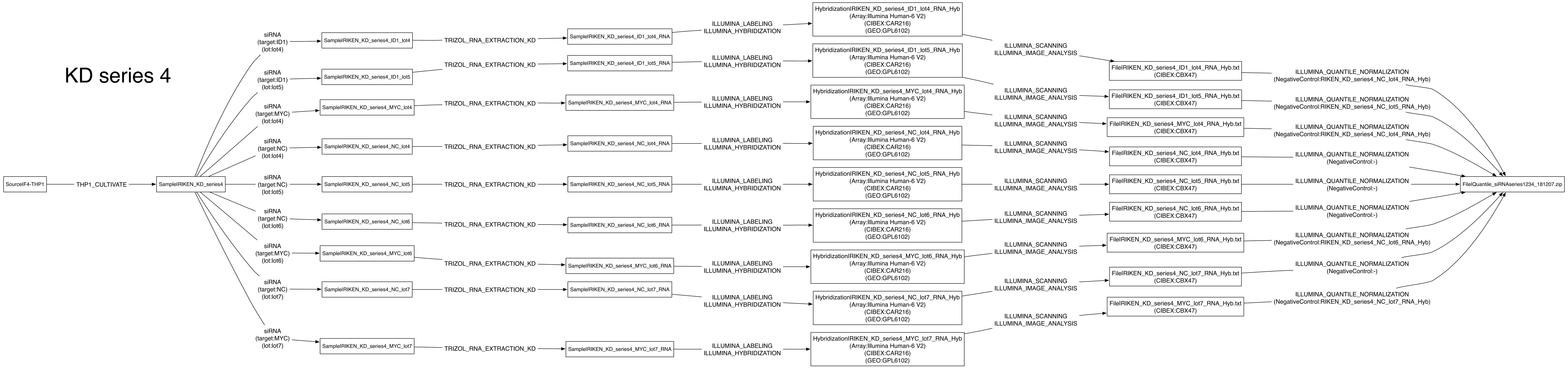
SourceIF4-THP1
THP1_CULTIVATE
SampleIRIKEN_KD_series4
siRNA (target:ID1) (lot:lot4)
SampleIRIKEN_KD_series4_ID1_lot4
TRIZOL_RNA_EXTRACTION_KD
SampleIRIKEN_KD_series4_ID1_lot4_RNA
ILLUMINA_LABELING ILLUMINA_HYBRIDIZATION
HybridizationIRIKEN_KD_series4_ID1_lot4_RNA_Hyb (Array:Illumina Human-6 V2) (CIBEX:CAR216) (GEO:GPL6102)
ILLUMINA_SCANNING ILLUMINA_IMAGE_ANALYSIS
FileIRIKEN_KD_series4_ID1_lot4_RNA_Hyb.txt (CIBEX:CBX47)
ILLUMINA_QUANTILE_NORMALIZATION (NegativeControl:RIKEN_KD_series4_NC_lot4_RNA_Hyb)
siRNA (target:ID1) (lot:lot5)
SampleIRIKEN_KD_series4_ID1_lot5
TRIZOL_RNA_EXTRACTION_KD
SampleIRIKEN_KD_series4_ID1_lot5_RNA
ILLUMINA_LABELING ILLUMINA_HYBRIDIZATION
HybridizationIRIKEN_KD_series4_ID1_lot5_RNA_Hyb (Array:Illumina Human-6 V2) (CIBEX:CAR216) (GEO:GPL6102)
ILLUMINA_SCANNING ILLUMINA_IMAGE_ANALYSIS
FileIRIKEN_KD_series4_ID1_lot5_RNA_Hyb.txt (CIBEX:CBX47)
ILLUMINA_QUANTILE_NORMALIZATION (NegativeControl:RIKEN_KD_series4_NC_lot5_RNA_Hyb)
siRNA (target:MYC) (lot:lot4)
SampleIRIKEN_KD_series4_MYC_lot4
TRIZOL_RNA_EXTRACTION_KD
SampleIRIKEN_KD_series4_MYC_lot4_RNA
ILLUMINA_LABELING ILLUMINA_HYBRIDIZATION
HybridizationIRIKEN_KD_series4_MYC_lot4_RNA_Hyb (Array:Illumina Human-6 V2) (CIBEX:CAR216) (GEO:GPL6102)
ILLUMINA_SCANNING ILLUMINA_IMAGE_ANALYSIS
FileIRIKEN_KD_series4_MYC_lot4_RNA_Hyb.txt (CIBEX:CBX47)
ILLUMINA_QUANTILE_NORMALIZATION (NegativeControl:RIKEN_KD_series4_NC_lot4_RNA_Hyb)
siRNA (target:NC) (lot:lot4)
SampleIRIKEN_KD_series4_NC_lot4
TRIZOL_RNA_EXTRACTION_KD
SampleIRIKEN_KD_series4_NC_lot4_RNA
ILLUMINA_LABELING ILLUMINA_HYBRIDIZATION
HybridizationIRIKEN_KD_series4_NC_lot4_RNA_Hyb (Array:Illumina Human-6 V2) (CIBEX:CAR216) (GEO:GPL6102)
ILLUMINA_SCANNING ILLUMINA_IMAGE_ANALYSIS
FileIRIKEN_KD_series4_NC_lot4_RNA_Hyb.txt (CIBEX:CBX47)
ILLUMINA_QUANTILE_NORMALIZATION (NegativeControl:-)
siRNA (target:NC) (lot:lot5)
SampleIRIKEN_KD_series4_NC_lot5
TRIZOL_RNA_EXTRACTION_KD
SampleIRIKEN_KD_series4_NC_lot5_RNA
ILLUMINA_LABELING ILLUMINA_HYBRIDIZATION
HybridizationIRIKEN_KD_series4_NC_lot5_RNA_Hyb (Array:Illumina Human-6 V2) (CIBEX:CAR216) (GEO:GPL6102)
ILLUMINA_SCANNING ILLUMINA_IMAGE_ANALYSIS
FileIRIKEN_KD_series4_NC_lot5_RNA_Hyb.txt (CIBEX:CBX47)
ILLUMINA_QUANTILE_NORMALIZATION (NegativeControl:-)
FileIQuantile_siRNAseries1234_181207.zip
siRNA (target:NC) (lot:lot6)
SampleIRIKEN_KD_series4_NC_lot6
TRIZOL_RNA_EXTRACTION_KD
SampleIRIKEN_KD_series4_NC_lot6_RNA
ILLUMINA_LABELING ILLUMINA_HYBRIDIZATION
HybridizationIRIKEN_KD_series4_NC_lot6_RNA_Hyb (Array:Illumina Human-6 V2) (CIBEX:CAR216) (GEO:GPL6102)
ILLUMINA_SCANNING ILLUMINA_IMAGE_ANALYSIS
FileIRIKEN_KD_series4_NC_lot6_RNA_Hyb.txt (CIBEX:CBX47)
ILLUMINA_QUANTILE_NORMALIZATION (NegativeControl:-)
siRNA (target:MYC) (lot:lot6)
SampleIRIKEN_KD_series4_MYC_lot6
TRIZOL_RNA_EXTRACTION_KD
SampleIRIKEN_KD_series4_MYC_lot6_RNA
ILLUMINA_LABELING ILLUMINA_HYBRIDIZATION
HybridizationIRIKEN_KD_series4_MYC_lot6_RNA_Hyb (Array:Illumina Human-6 V2) (CIBEX:CAR216) (GEO:GPL6102)
ILLUMINA_SCANNING ILLUMINA_IMAGE_ANALYSIS
FileIRIKEN_KD_series4_MYC_lot6_RNA_Hyb.txt (CIBEX:CBX47)
ILLUMINA_QUANTILE_NORMALIZATION (NegativeControl:RIKEN_KD_series4_NC_lot6_RNA_Hyb)
siRNA (target:NC) (lot:lot7)
SampleIRIKEN_KD_series4_NC_lot7
TRIZOL_RNA_EXTRACTION_KD
SampleIRIKEN_KD_series4_NC_lot7_RNA
ILLUMINA_LABELING ILLUMINA_HYBRIDIZATION
HybridizationIRIKEN_KD_series4_NC_lot7_RNA_Hyb (Array:Illumina Human-6 V2) (CIBEX:CAR216) (GEO:GPL6102)
ILLUMINA_SCANNING ILLUMINA_IMAGE_ANALYSIS
FileIRIKEN_KD_series4_NC_lot7_RNA_Hyb.txt (CIBEX:CBX47)
ILLUMINA_QUANTILE_NORMALIZATION (NegativeControl:-)
siRNA (target:MYC) (lot:lot7)
SampleIRIKEN_KD_series4_MYC_lot7
TRIZOL_RNA_EXTRACTION_KD
SampleIRIKEN_KD_series4_MYC_lot7_RNA
ILLUMINA_LABELING ILLUMINA_HYBRIDIZATION
HybridizationIRIKEN_KD_series4_MYC_lot7_RNA_Hyb (Array:Illumina Human-6 V2) (CIBEX:CAR216) (GEO:GPL6102)
ILLUMINA_SCANNING ILLUMINA_IMAGE_ANALYSIS
FileIRIKEN_KD_series4_MYC_lot7_RNA_Hyb.txt (CIBEX:CBX47)
ILLUMINA_QUANTILE_NORMALIZATION (NegativeControl:RIKEN_KD_series4_NC_lot7_RNA_Hyb)

Time course ChIP/chip

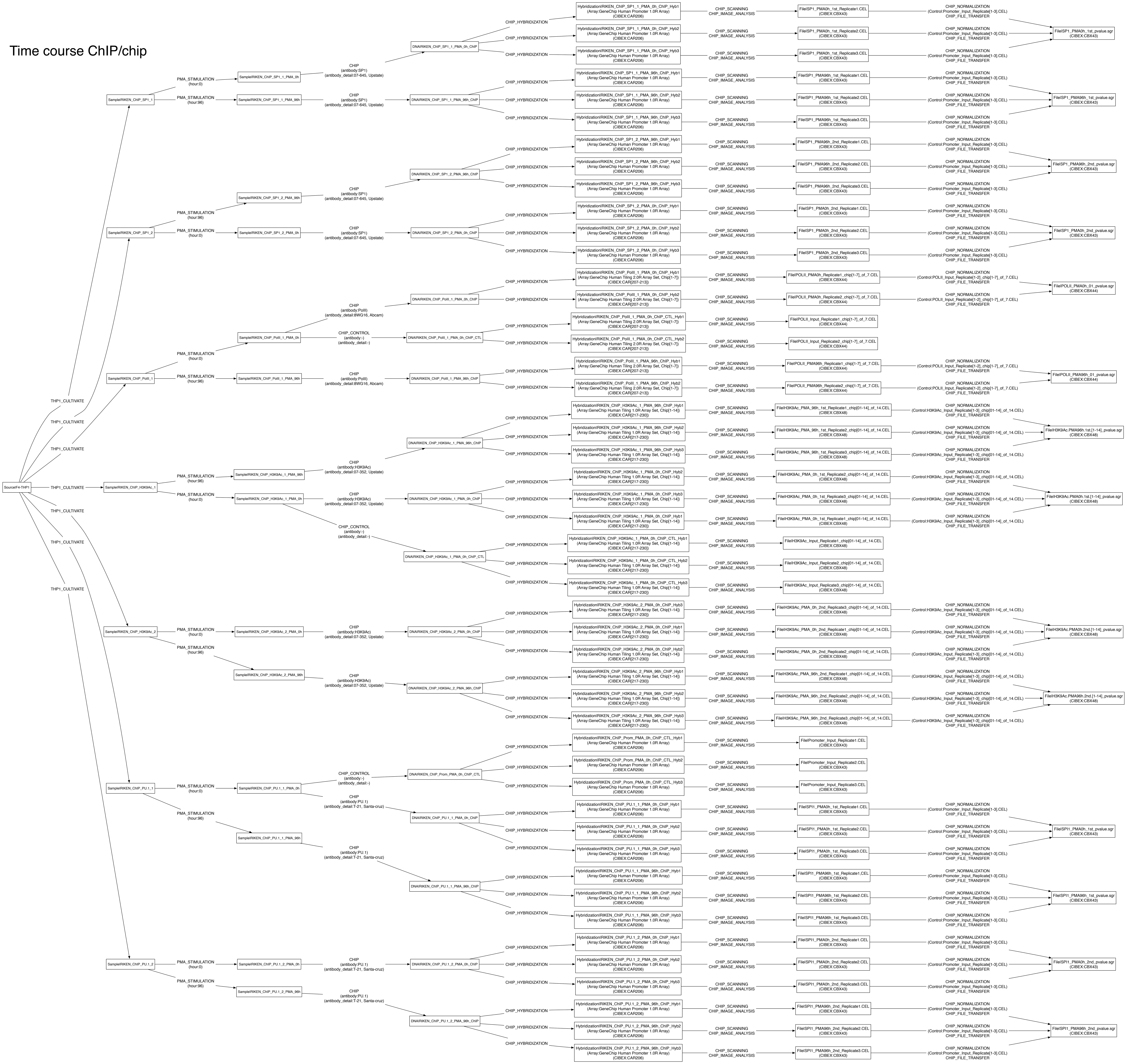

Time course expression (CAGE, Microarray)

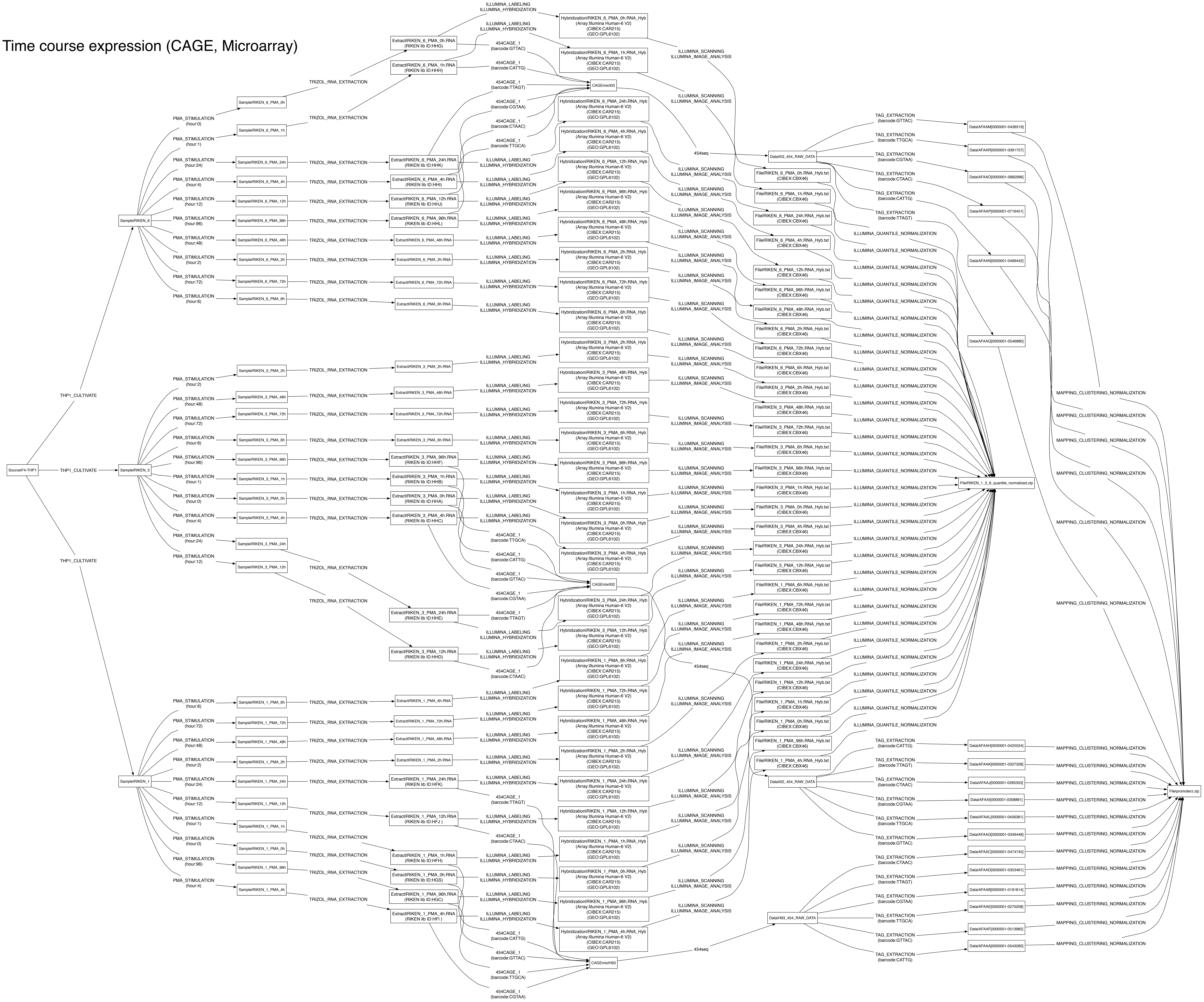

Supplement: Additional file 3 — SDRF2GRAPH representation for the FANTOM4 data. SDRF2GRAPH output of the additional file 2. [file 1471-2105-10-133-S3.pdf]
